# Single specimen genome assembly of *Culicoides stellifer* shows evidence of a non-retroviral endogenous viral element

**DOI:** 10.1186/s12864-025-11449-5

**Published:** 2025-03-14

**Authors:** Jessica Castellanos-Labarcena, Yoamel Milián-García, Tyler A. Elliott, Dirk Steinke, Robert Hanner, Sarah J. Adamowicz

**Affiliations:** 1 Department of Integrative Biology, University of Guelph, 50 Stone Rd E, Guelph, ON N1G 2W1 Canada; 2 Centre for Biodiversity Genomics, University of Guelph, 50 Stone Rd E, Guelph, ON N1G 2W1 Canada

**Keywords:** *Culicoides*, Vesicular stomatitis virus, Genome assembly, Vector, Arboviruses

## Abstract

**Background:**

Advancing our knowledge of vector species genomes is a key step in our battle against the spread of diseases. Biting midges of the genus *Culicoides* are vectors of arboviruses that significantly affect livestock worldwide. *Culicoides stellifer* is a suspected vector with a wide range distribution in North America, for which cryptic diversity has been described.

**Results:**

With just one specimen of *C. stellifer*, we assembled and annotated the nuclear and mitochondrial genome using the ultra-low input DNA PacBio protocol. The genome assembly is 119 Mb in length with a contig N50 value of 479.3 kb, contains 11% repeat sequences and 18,895 annotated protein-coding genes. To further elucidate the role of this species as a vector, we provide genomic evidence of a non-retroviral endogenous viral element integrated into the genome that corresponds to rhabdovirus nucleocapsid proteins, the same family as the vesicular stomatitis virus.

**Conclusions:**

This genomic information will pave the way for future investigations into this species’s putative vector role. We also demonstrate the practicability of completing genomic studies in small dipterans using single specimens preserved in ethanol as well as introduce a workflow for data analysis that considers the challenges of insect genome assembly.

**Supplementary Information:**

The online version contains supplementary material available at 10.1186/s12864-025-11449-5.

## Background

*Culicoides* (Diptera: Ceratopogonidae) are among the most important vectors of arboviruses pathogenic to livestock and wildlife. The genus is highly diverse, with 1,347 valid species [1], of which 151 are currently recognized in North America, occupying a broad geographical range [2]. Here, *C. sonorensis* Wirth and *C. insignis* Lutz are the only species with confirmed vector status and they are known to transmit bluetongue virus [BTV], vesicular stomatitis virus (VSV), and epizootic hemorrhagic disease virus [EHDV] [3]. Reports of increased rates of BTV and EHDV outside of the geographic range of both species suggest that there might be an expansion or shift in species distribution due to climate change, or other species not recognized as vectors could be involved [4, 5]. One such putative vector species is *C. stellifer* Coquillett, abundant and widely distributed in the United States of America (USA) and eastern Canada. Several field-collected individuals in the USA have been confirmed to carry arboviruses, but it has been challenging to complete vector competence assays [3]. *Culicoides stellifer* has been closely associated with ungulate species, although host associations for many Nearctic species are poorly understood [6].

Despite the serious threat to animal health these vectors represent, and the significant economic losses outbreaks could cause, there is a lack of genomic studies of *Culicoides*, as well as little understanding of the systematics of the group [1, 4, 7]. The genome assembly of only two species is available in NCBI; *C. sonorensis* (GCA_900258525.3) [7, 8] and *C. brevitarsis* Kieffer (GCF_036172545.2). Partial or complete annotated mitogenomes, which are a valuable resource for studying the phylogenetics and systematics, are available for only four species (*C. arakawae* Arakawa, *C. sonorensis*,* C. brevitarsis* and *C. biguttatus* Coquillett) [9]. Genomic information is critical for understanding the unique evolutionary features of this group, phylogenetic relationships, vector competency for arboviruses, and cryptic diversity [3, 7, 9]. One of the main causes that only a limited amount of *Culicoides* genomes have been sequenced in is perhaps the difficulty to obtain sufficient quantities of high molecular weight DNA. Species are small, < 3 mm body length, which typically generates very low concentration DNA extracts from single specimens (5 to 43 ng) [9].

Advances in long-read sequencing technologies that allow low amounts of input material and modifications to increase starting DNA concentration for library preparation have opened the door to generating high-quality genome assemblies for small arthropods [10]. Particularly, the PacBio HiFi ultra-low DNA input workflow starts with as low as 5 ng genomic DNA for whole-genome amplification and is recommended for genome sizes of up to 500 Mb. This workflow was used to generate a *de novo* genome assembly for *Drosophila melanogaster* [11] and two submillimeter Collembola species (*Desoria tigrina* and *Sminthurides aquaticus*) [12]. It allows sequencing the genome from a single, field-preserved specimen, generating medium-size fragments (10–25 kb) with high base accuracy (99.8%), which can be used to produce assemblies that are more contiguous and with a higher base accuracy.

The expansion of *Culicoides*-borne pathogens in Eastern Canada, especially in Ontario, highlights the need to characterize potential vectors, viruses and hosts. *Culicoides stellifer* is suspected to represent a species complex, with cryptic diversity reported for samples collected in Ontario [13]. In this study we present a genome assembly of a *C. stellifer* specimen collected in Southern Ontario. In an attempt to provide more supporting evidence that this species may transmit one or more RNA viruses, we set out to query the genome for viral fragments, also known as non-retroviral endogenous viral elements (nrEVE) of BTV, EHDV, VSV and West Nile virus (WNV) viruses [14, 15, 16]. This phenomenon is known as virus-to-host horizontal gene transfer and is associated with persistent viral infection [17]. Given the complexity of *Culicoides* pathogens, crypticity, and unknown vector species, we developed a methodology and a bioinformatics pipeline to generate key genomic information for this group. This will significantly contribute to identifying new vector species, understanding the phylogenetic relationships of the group, and evolutionary processes involved in vector competence across Diptera.

## Methods

### Sample collection and genome sequencing

**Fig. 1:**
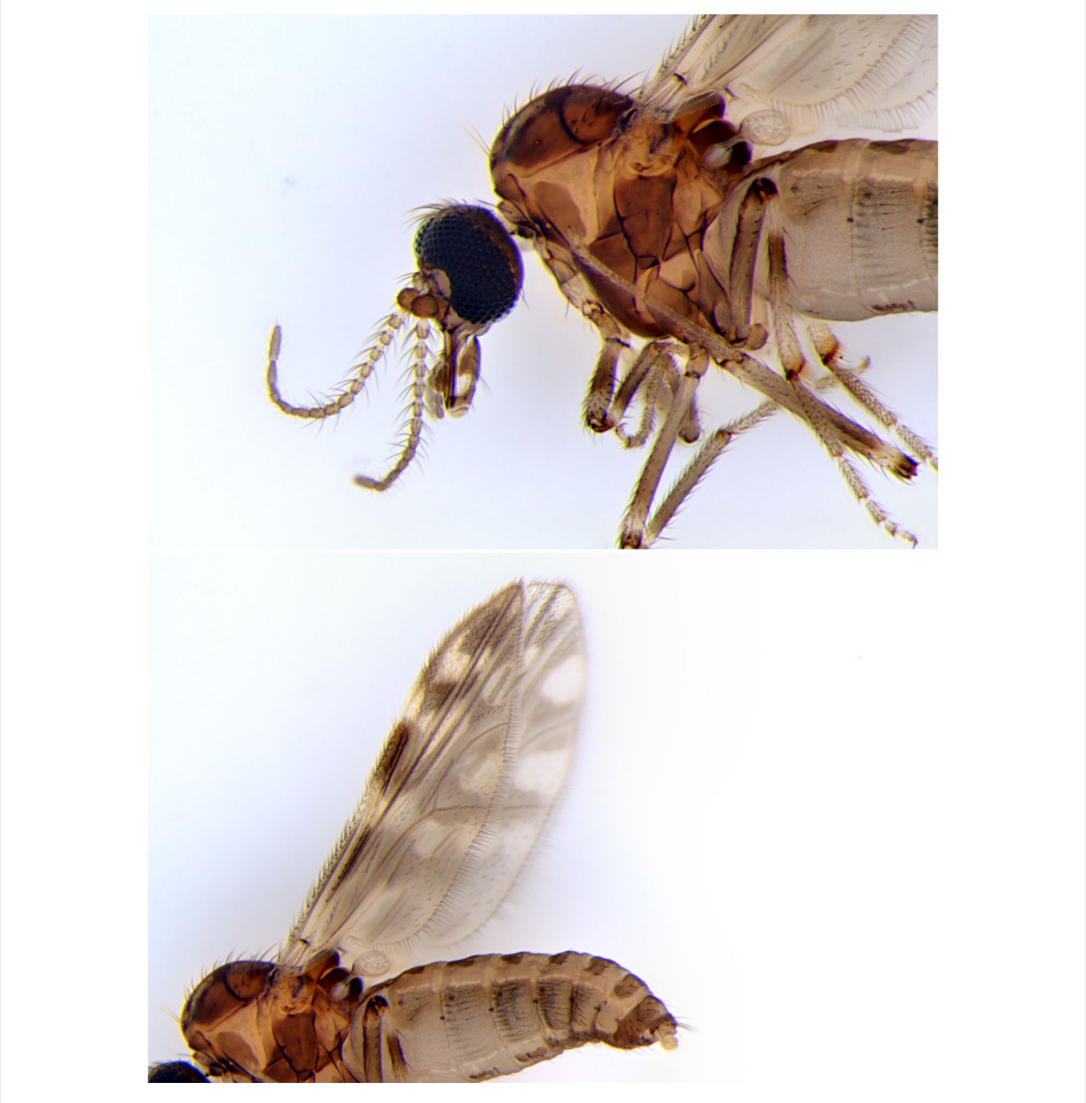
Images of the *Culicoides stellifer* specimen used to generate the genome assembly, highlighting the wing patterns. (Photo by Kate Lindsay)

*Culicoides stellifer* specimens were collected at the Ontario Veterinary College Dairy Barn at the University of Guelph, Ontario, Canada, using miniature Centre for Disease Control (CDC) UV light traps (Bioquip, CA, USA). The specimens were identified using the dichotomous key for *Culicoides* of Ontario [5]. Images were taken using the Leica MC170 HD Camera mounted on a Leica M205 A microscope (Leica Microsystems Wetzlar, Germany) (Fig. 1). Five female individuals preserved in 95% ethanol were sent to the University of Delaware’s DNA Sequencing & Genotyping Center in Newark, DE, USA. As *Culicoides* species are less than 3 mm long and weigh < 1 mg, we decided to use the ultra-low DNA Input protocol from PacBio [11] to generate genomic data from a single specimen. Genomic DNA was extracted from each individual separately using the MagAttract HMW DNA kit (Qiagen). DNA quantification was completed using a Qubit Fluorimeter, and DNA fragment sizes were assessed by a Femto Pulse system (Agilent) for fragments of a length around 12–14 kb. The amount and quality of genomic DNA for only one individual was sufficient to move forward with library preparation.

SMRTbell gDNA was constructed following the protocol “Preparing HiFi SMRT-bell libraries from Ultra-Low DNA input” using the SMRTbell Express Template Prep Kit 3.0 (Pacbio, 102-182-700). After a BluePippin size selection (Sage Science, PAC20KB) at 6 kb, the average library size was 10 kb measured on a Femto Pulse system Agilent). Sequencing was performed on a SMRT 8 M cell on the Sequel IIe using the Sequel II Binding kit 2.2/Sequel II Sequencing kit 2.0 with a 30-hours movie.

### Preassembly processing

PacBio Hifi reads were first processed to trim PCR adapter sequences and to remove PCR duplicates. We used the *lima* for PCR adapter trimming and *pbmarkdups* for PCR duplicate removal, both available in *pbbioconda* (https://github.com/PacificBiosciences/pbbioconda*).* Properties of the genome, such as genome size, levels of heterozygosity and repeat content, were estimated by analysis of *K*-mer frequencies. We used Meryl v1.4.1, as implemented in Merqury v1.3 [18] and used the size of the *C. sonorensis* genome as a reference [7] to estimate the *k*-mer size to use. Frequencies of *k*-mers (*K* = 19) were counted using Meryl v1.4.1. With the *k*-mer histogram, we estimated the genome properties using GenomeScope v2.0 [19].

### Mitogenome assembly and annotation

For the assembly of the mitochondrial genome, we used MitoHiFi v3.2 [20], starting with the raw reads. The first assembled mitogenome was significantly larger than expected, so we decided to use only reads mapped to the reference genome (*C. arakawae* ) and assembled the mitogenome using Pacific Biosciences’ Improved Phase Assembly (IPA, v1.8.0) HiFi Genome Assembler pipeline (https://github.com/PacificBiosciences/pbipa*).* We annotated the mitogenome using MITOS2 v2.1.8 as implemented in the Galaxy workbench [21].

### Genome assembly

Genome assembly was conducted after removing the mitochondrial genome reads. We used two assemblers, IPA v1.8.0 and Hifiasm v0.16.0 [22]. For Hifiasm, we used different similarity thresholds for duplicate haplotypes to be purged (-s parameter) following the author’s recommendations (s = 0.75, s = 0.55, and s = 0.35). The overall quality of these preliminary assemblies, especially continuity and completeness, was estimated using assembly-stats v17.02 (rjchallis/assembly-stats 17.02) and Benchmarking Universal Single-Copy Orthologs (BUSCO) v5.6.1 [23] with a Diptera database (diptera_odb10.gz). Given the high level of duplication of preliminary assemblies and the large size of genomes compared to the predicted value, we conducted *a posteriori* purging of duplicates using *purge_dups* [24]. The resulting assemblies showed similar characteristics in terms of contiguity and completeness; we selected the assembly generated with Hifiasm -s 0.35 for subsequent analyses as it has the largest N50 value. To further evaluate the quality of the assembly, we used Merqury v1.4.1 [18] to estimate base-level accuracy and completeness as well as BlobToolkit for contamination identification and isolation [25].

### Repeat element annotation

We annotated transposable elements (TE), satellite DNA, simple and low-complexity repeats using Earl Grey v.4.1.1 [26]. Via Earl Grey, we used RepeatMasker v.4.1.6 [27] to identify and mask simple and low-complexity repeats, along with the Diptera subset of repeats from the growing, open source repeat reference library Dfam v.3.7 [28]. Once masked for these repeats, the genome was analyzed with RepeatModeler2 v.2.0.5 [29] for de novo repeat identification and classification. Earl Grey next employed a BLAST-extract-align-trim procedure on each repeat consensus sequence to refine their boundaries and improve the quality of the reference library, along with clustering of consensus sequences using CD-HIT to reduce redundancy [30, 31]. Next, LTR_FINDER [32, 33] was used to further detect any missing long terminal repeat (LTR) retrotransposons before combining all collected repeats and masking and annotating the genome once more with RepeatMasker. Finally, Earl Grey used RepeatCraft [34] to merge physically close or overlapping repeat fragments in the annotation which have the same classification. The library of generated consensus sequences was translated into open reading frames of at least 300 bp in all six frames using getorf [35], and these were queried against the Pfam v.35.0 [36] protein reference library using pfam_scan.pl to detect instances of host gene contamination in the repeat reference library. The output was manually inspected due to the small size of the reference library, and 22 consensus sequences were removed from the library.

To provide additional evidence for the proper classification of TEs, the tool TEsorter v1.4.6 [37] was employed to extract open reading frames from all reference sequences, query them using hmmscan against compiled protein reference libraries of terminal inverted repeat (TIR) DNA transposons [38], long interspersed nuclear elements (LINE) [39] and LTR retrotransposons [40]. Due to the large proportion of unknown repeats, in terms of the number consensus sequences and percentage of total repeats annotated, all RepeatModeler2 consensus sequences of at least 100 bp and covering at least 10,000 in the assembly were manually inspected. For each consensus sequence, this involved one or more of the following steps recommended by Goubert et al. [41]: (1) use of TE_ManAnnot to extract blast hits for each consensus that were at least half the size of the consensus, along with enough flanking DNA to resolve the termini of the given consensus, (2) alignment of all hits using MAFFT v7.453 [42] to accommodate the high frequency of indels in repeats, (3) the removal of gaps in the alignment where 80% of the sequences featured a gap via T-COFFEE v13.46.0 [43], (4) the inspection of the alignment to confirm the consensus sequence did not need to be extended or adjusted, (5) the creation of a new consensus sequence when needed via cons in EMBOSS, and (6) the use of TE-Aid to visualize the size and number of hits of a given consensus, the divergence of hits from the consensus, the presence of repetitive structures within the consensus, and the presence of TE coding regions via blastp to the RepeatMasker RepeatPeps protein database.

If needed, consensus sequences were re-classified based upon the evidence accumulated in this final curatorial step. In the EarlGrey file structure, we deleted the contents of the mergedRepeats folder, and replaced the *-families.fa.strained in the *-strained folder with the final curated repeat library. EarlGrey was then run again to restart the pipeline at the final RepeatMasker and RepeatCraft steps to generate a final repeat annotation.

### Gene prediction and functional annotation

We completed the gene prediction on the soft-masked genome assembly using the BRAKER3 v3.0.8 pipeline [44], providing protein homology information as extrinsic evidence. We used the Arthropoda clade-partitioned file of OrthoDB 11 [45] as the source of reference protein sequences. We functionally annotated the predicted protein-coding genes using DIAMOND BLASTP [46], searching against the Swiss-Prot protein database 2024_02 (https://www.uniprot.org/*).* We filtered the output for E-value < 1e-10 and sequence identity > 30%. The predicted genes were also mapped to Kyoto Encyclopedia of Genes and Genomes (KEGG) pathways to classify functional categories using BlastKOALA (https://www.kegg.jp/blastkoala/*).* Additionally, we ran InterProScan v5.67-99.0 [47] with all default settings and added the option of looking for the Gene Ontology (GO) annotation.

### Non-retroviral endogenous viral identification

Nucleotide sequences for EHDV, VSV and WNV viruses were downloaded from GenBank, and the curated set of BTV sequences from BTV-GLUE [48]. Incomplete and artificial sequences were filtered out along with VSV and WNV viruses shorter than 10,000 bp by data processing in R v.4.3.2 [49], aided by tidyverse v2.0.0 [50], Biostrings v2.70.2 [51] and seqRFLP v1.0.1 (https://github.com/helixcn/seqRFLP). EHDV and BTV are viruses with segmented genomes, so each segment was detected and sorted before multiple sequence alignments were built for each viral segment or whole virus for the others, using MUSCLE [52] and default settings. A hidden Markov model (HMM) was generated for each alignment using hmmbuild in HMMER3 [53], and the *C. stellifer*, *C. sonorensis* (GCA_900258525.3) and *C. brevitarsis* (GCA_036172545.2) assemblies were queried against each of these models using nhmmer, along with the raw reads used in creating the *C. stellifer* assembly.

## Results

### Hifi sequencing with ultra-low DNA input workflow

**Fig. 2:**
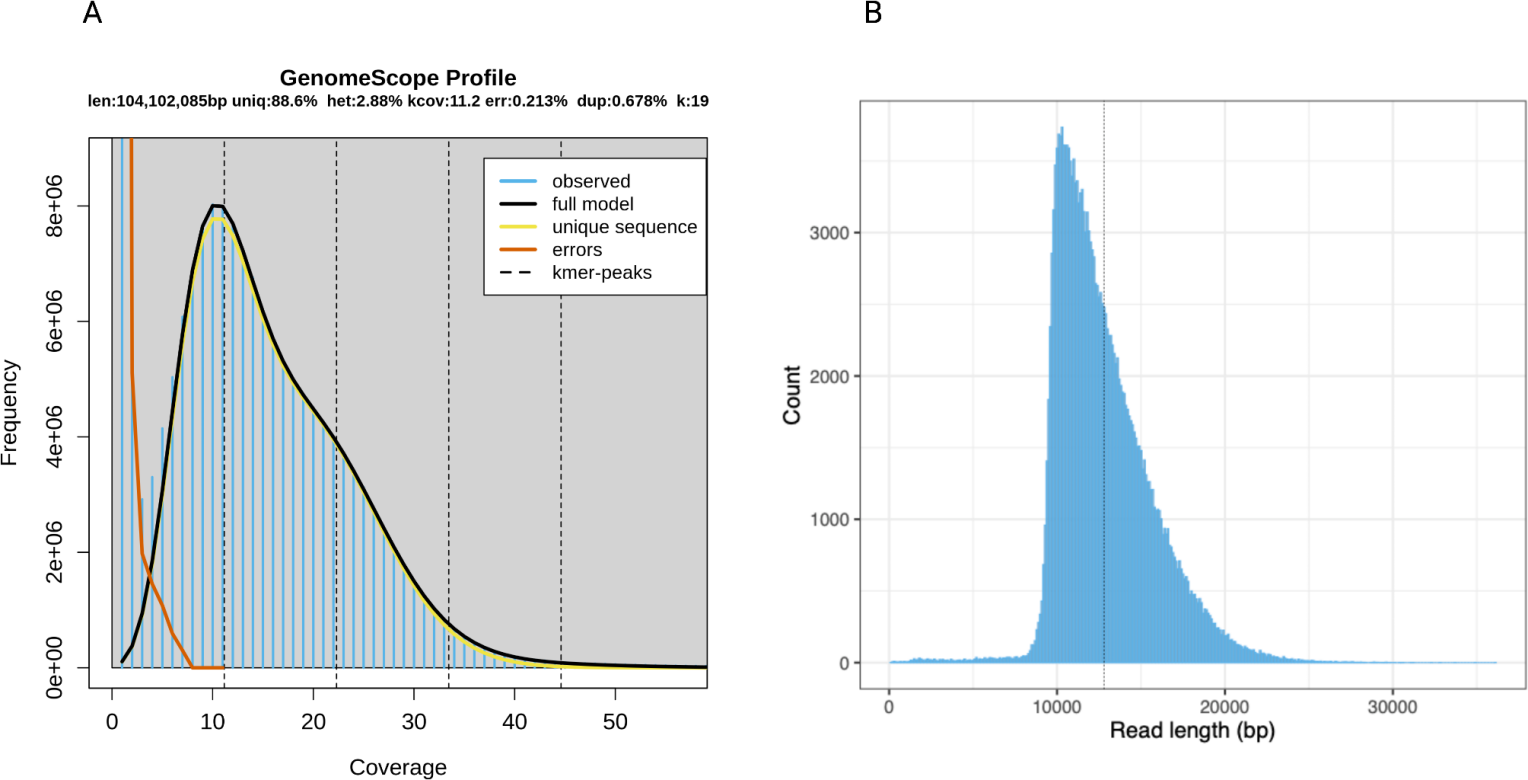
Genome properties based on raw data exploration. (**A**) GenomeScope results in linear coordinates on the PacBio Hifi sequencing dataset for one individual of *C. stellifer.* The genome size (len) is predicted to be around 104 Mb, and 88.6% of the 19-mers are unique (aa), suggesting that the genome has around 11% repetitive content. Heterozygosity (ab), mean k-mer coverage for heterozygous bases (kcov), read error rate (err), the average rate of read duplications (dup), k-mer size used in the run (k: ), and ploidy (p: ) is also reported. The sequencing errors are identified by low-coverage k-mers. (**B**) Frequency histogram of the read length for the PacBio Hifi sequencing dataset for one individual of *C. stellifer*. The dashed lines represent the mean value

The ultra-low DNA input protocol includes a PCR amplification step to generate sufficient material for sequencing. This was a critical consideration when selecting this workflow to generate high-quality genomic information from a single *C. stellifer* specimen. PCR products ranged from 5 to 8 kb. These values suggest that the gDNA had some degree of fragmentation and that short fragments were preferentially amplified. Sequencing output resulted in 191,906 PacBio Hi-Fi reads with an average read length of ~ 13,000 bp and 20X coverage. The genome size was estimated to be approximately 104 Mb, with a heterozygosity of 2.88% and 11.4% of repeat sequences (Fig. 2).

### Mitogenome assembly

Long-read sequencing technologies for mitochondrial genome assembly in *Culicoides* haven’t been explored before. We started by using the MitoHifi toolkit for mitochondrial assembly from Hifi data. The pipeline failed to correctly assemble the mitochondrial genome, as it generated a molecule much larger than expected (~ 50,000 bp). It is likely that the misassembly might be related to shorter reads, insufficient coverage, or the presence of nuclear-mitochondrial DNA (NUMTs). We selected 128 reads that mapped to a reference mitogenome (*C. arakawae*) and generated a *de-novo* assembly for *C. stellifer’s* mitochondrial genome using IPA assembler. This resulted in a 16,607 bp mitochondrial genome, which is within the range of mitogenome lengths previously reported for other species of the genus [9, 54].

**Fig. 3:**
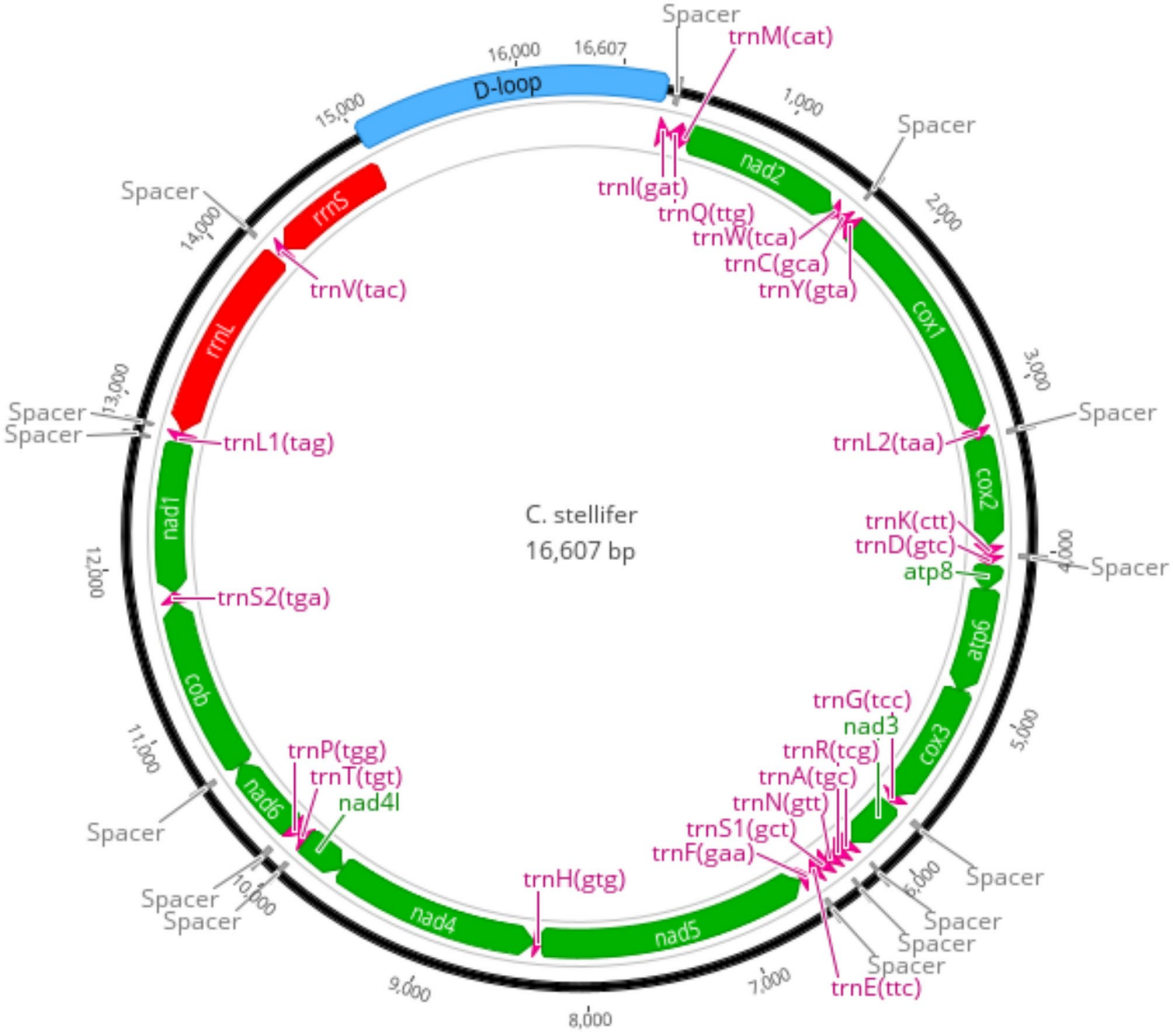
Mitochondrial genome annotation for *C. stellifer.* Protein-coding genes, rRNA, and tRNA are represented in green, brown and orange, respectively. The control region (D-loop) is marked in blue, and the intergenic spacers are marked in red. The annotation was completed using MITOS2 v2.1.8, and the figure was generated using Geneious prime v11:08

The annotation using MITOS2 identified 13 protein-coding genes (PCGs), 22 transfer RNAs (tRNA), and two ribosomal RNAs (rRNA) (Fig. 3). The assembly was circularized and overall it showed the same gene arrangement previously described for other species in the genus. PCGs sizes ranged from 152 (ATP8) to 1,732 bp (NAD5). Transfer RNA sizes ranged from 48 [tRNA F(gaa) to 72 bp [tRNA V(tac)], while rRNA lengths were 781 (rRNA S) and 1,284 bp (rRNA L). The estimated control region size was 1,842 bp. Additionally, 17 spacers were identified, ranging in size from 1 to 18 bp.

### Genome assembly

We compared two long-read assembly tools (IPA and Hifiasm) and various levels of duplicate purging in Hifiasm (-s parameter). Overall, Hifiasm produced the best assemblies. All four initial assemblies resulted in primary genomes with a size > 146 Mb and almost 30% of complete-duplicated genes reported by BUSCO. Different values of the similarity threshold for duplicate haplotigs (- s parameter) in the Hifiasm assemblies resulted in a slight decrease in the total number of contigs and an increase in the N50, producing overall similar values (Table 1).

**Table 1:** Summary statistics of the *C. stellifer* primary genome assembly using hifiasm compared to two other genomes of the genus available in NCBI

Genome Assembly	*C. stellifer* HiFiasm -s 0.35 purge_dups	*C. sonorensis* Velvet (GCA_900258525.3)	*C. brevitarsis* Raven; Polca (Masurca); Racon.
Sequencing technology	PacBio Hifi	Illumina HiSeq	Oxford Nanopore PromethION; Illumina NovaSeq
**Genome statistics**			
Total length (Mb)	119	155.9	129.5
Number of contigs	450	3858	223
Number of scaffolds	0	0	149
Longest contig or scaffold (bp)	1,731,461	763,582	46,604,242
Mean contig or scaffold length (bp)	265,155	40,420	863,398
N50	479,265	109,184	3.5 Mb
N90	132,711	NA	NA
L50	81	395	NA
L90	261	NA	NA
GC content	30.8%	28.3%	27.9%
**Total BUSCO for the genome assembly**			
Complete BUSCO	2953 (89.9%)	2913 (88.7%)	91.9%
Complete single copy	2882 (87.7%)	2502 (76.2%)	89.3%
Complete duplicated	71 (2.2%)	411 (12.5%)	2.6%
Fragmented	51 (1.6%)	66 (2.0%)	0.6%
Missing	281 (8.5%)	306 (9.3%)	7.5%

**Fig. 4:**
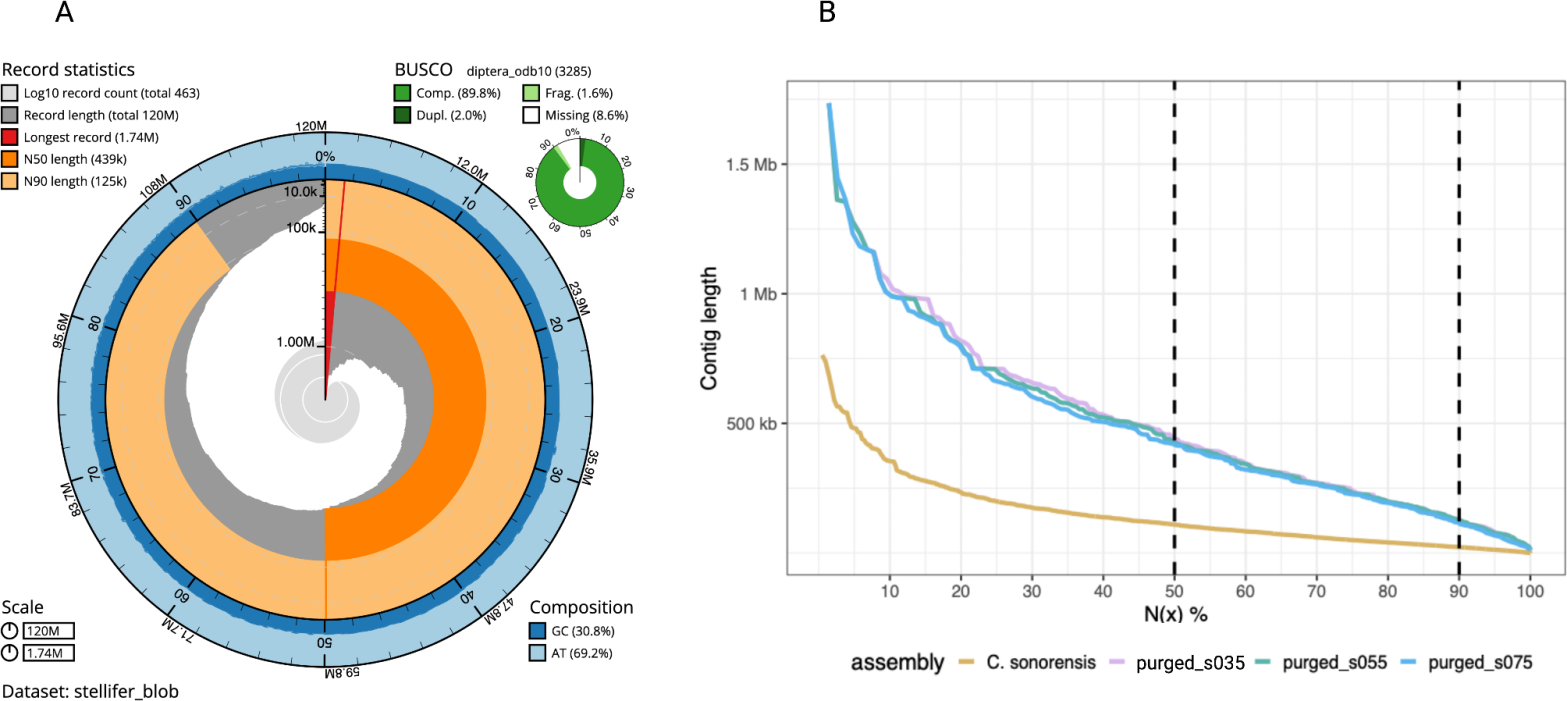
Contig-level assembly of *C. stellifer.* (**A**) Snail plot showing lengths of all contigs. The longest contig is represented in red, N50 in dark orange, and N90 in light orange. The outer ring shows the GC content of the genome. (**B**) Visualization of assembly contiguity showing contig sizes on the Y-axis for which x percent of the assembly consists of contigs of at least that size. The three assemblies of *C. stellife*r with various levels of similarity purging are compared to the assembly of *C. sonorensis*

Purging duplicated regions with purge_dups reduced the number of contigs by half, increased contiguity and significantly decreased the number of duplicated genes reported by BUSCO (Table 1). The 119 Mb size assembled genome is closer to the estimate of 105 Mb obtained from the k-mer analysis. In comparison to the other two available *Culicoides* genomes, our assembly displays good quality in terms of contiguity (N50 and L50) and completeness (Fig. 4). The BUSCO scores of our assemblies (89.8% complete (C) BUSCOs (including 2.0% duplicated [D]), 1.5% fragmented (F), and 8.6% missing (M)) are very similar to those of the genome of *C. brevitarsis*, whose assembly includes three chromosomes and unplaced scaffolds. The methodology presented in our study overcomes many challenges faced in generating the genome of *C. sonorensis*, as the latter involved pooling many individuals using short-read sequencing.

As our final assembly, we selected the one with the highest N50 and the lowest number of duplicated BUSCOs without significantly decreasing the complete BUSCO score. The genome assembly (referred to as purged_s030) comprises 450 contigs, totalling 119,322,097 bp, contig N50 of 479,264 bp and L50 of 81 (Fig. 4). We estimated a high base accuracy (QV = 53.3) and 90% completeness based on the k-mer comparison between the assembly and those found in the PacBio raw reads.

### Genome annotation

Overall, the degree of repetitive content in the genome assembly of *C. stellifer* was approximately 15 Mb of repetitive elements, representing 11% of the genome assembly (Table 2). Initially, nearly half of all repeats were classified as unknown. Due to the small size of this reference library, we decided to manually investigate the largest and most abundant consensus sequences. Many of the unknown repeats were determined to be non-autonomous TIR DNA transposons, and in general, all DNA transposons were characterized by a lack of substantial coding regions for transposases. In an attempt to find autonomous elements, the repeat library output from a larger version of the assembly with less purged duplicates (HiFiasm -s 0.75) was inspected for novel consensus sequences, and these were added to the existing repeat library and the genome was re-annotated. In this new library, a total of 4 consensus sequences of DNA transposons (TcMar-Tc1, TcMar-Tigger, TcMar-ISRm11, hAT-Tip100) had partial coding regions, but none of these appear to be functional.

**Table 2:** Summary of repeat elements annotated in the *C. stellifer* assembly. The numbers of consensus sequences in parentheses represent those generated by RepeatModeler2

Repeat	Superfamily	Base pairs	Consensus Sequences
**DNA transposon**			
TIR	Non-Autonomous	2,563,172	66 (59)
	hAT	496,695	9 (8)
	Tc1/Mariner	103,081	9 (3)
	piggyBac	55,206	1 (1)
	Other TIR	4,132	12
	*Total DNA*	3,222,286	97 (71)
**Retrotransposon**			
LTR	Bel-Pao	202,125	41 (5)
	Ty1/Copia	100,586	20 (3)
	Ty3-like	87,488	54 (2)
	Unclassified LTR	48,094	2 (2)
	*Total LTR*	423,038	117 (12)
LINE	I	239,478	30 (5)
	Unclassified LINE	121,740	4 (4)
	CR1	91,715	26 (6)
	R2	48,480	1 (1)
	RTE	27,718	4 (3)
	*Total LINE*	529,131	65 (19)
	*Total Retrotransposon*	952,169	182 (31)
	**Total TE**	4,174,455	279 (102)
**Other Repeats**			
Satellite/Simple/Low complexity		6,107,679	2976 (55)
Unknown		5,434,496	216 (216)
	**Total Repeats**	15,716,630	3443 (373)

Comparison and selective melding of the two libraries added new consensus sequences for four LTR retrotransposons with coding regions and well-resolved termini, as well as several LINE elements including an R2 consensus sequence. Retrotransposons make up a smaller fraction of the genome than DNA transposons, which stands in contrast to the pattern seen in the *C. sonorensis* genome [7]. Caution should be taken when comparing the repeats in these two genomes, as the methods differed, and the repeat annotation in the *C. sonorensis* assembly was not as thorough as was done for *C. stellifer*. In general, the *C. stellifer* assembly has a lower repeat content than *C. sonorensis* (~ 11% vs. 29.7%); however, this is not surprising when that repeat content is positively correlated with genome or assembly size [55].

A breakdown of the contribution of different components of Earl Grey to the resultant repeat library is useful when considering repeat annotation in novel genomes (Table 3.). Dfam is a growing, open-source database of repeats, and its current subset of Dipteran repeats stems from species distantly related to *C. stellifer*, hence the limited contribution to the annotation. Rather than being an indictment of Dfam, this stresses the value of submitting consensus sequences to Dfam to increase its taxonomic scope and useability for new genomes.

**Table 3:** Comparative statistics of repeat sequences detected by various sources and their annotation in the assembly

Repeat Source	Consensus Sequences	Mean Coverage/Consensus (bp)	Total Coverage (bp)
Dfam Diptera	181	1610	291,385
RepeatMasker	2891	966	2,792,913
RepeatModeler2	373	33,644	12,549,446

BRAKER3 predicted 18,895 proteins in the nuclear genome with 18,662 unique sequences. We annotated 10,524 proteins (55.7%) by searching against the Swiss-Prot protein sequence database. 7,283 genes were mapped to KEGG pathways using BlastKOALA (Table 4). Collectively, 7812 proteins were functionally annotated by InterProScan, of which 4057 were assigned a GO term. This resource provides complementary levels of protein annotation, including curated InterPro entries annotated with a unique name and GO terms. The following analyses were included in the output file: PANTHER, CATH-Gene3D, PROSITE Profiles, Pfam, SUPERFAMILY, SMART, FunFam, Conserved Domains Database (CDD), PRINTS, Hamap, PIRSF, NCBIfam and the Structure-Function Linkage Database (SFLD). These represent protein signature databases included in InterPro [56] that were scanned in an integrated way to predict protein functions and for which a match was found. Some of the results of these analyses are included in Table 4.

We annotated more than 3,000 additional protein-coding genes for either the *C. sonorensis* (15,612) or the *C. brevitarsis* (11,137) genome, respectively. This indicates that our workflow recovered a more complete set of genes for this group. We ran BUSCO in protein mode on the predicted proteins using the diptera_odb10 lineage dataset, which resulted in 91.5% complete BUSCO, including 8.3% duplicated, 1.0% fragmented and 7.5% missing. These values are similar to the report of *C. brevitarsis* (GCF_036172545.1-RS_2024_03) except for the complete and duplicated genes for which we report a slightly higher value (2.6% for *C. brevitarsis*). This difference is explained by the larger number of proteins predicted by BRAKER2 in our assembly compared to the annotation of *C. brevitarsis* using the NCBI Eukaryotic Genome Annotation Pipeline.

**Table 4:** Functional annotation of *C. stellifer* proteins

Genome annotation	Number of elements	Percentage
Predicted protein-coding genes (BRAKER2)	18,895	
Swiss Prot	10,524	55.7
KEGG (BlastKOALA)	7,342	38.9
Pfam	6,209	32.9
InterPro	6,807	36.0
GO	6,026	31.9

### Non-retroviral integrated RNA virus fragment identification

**Fig. 5:**
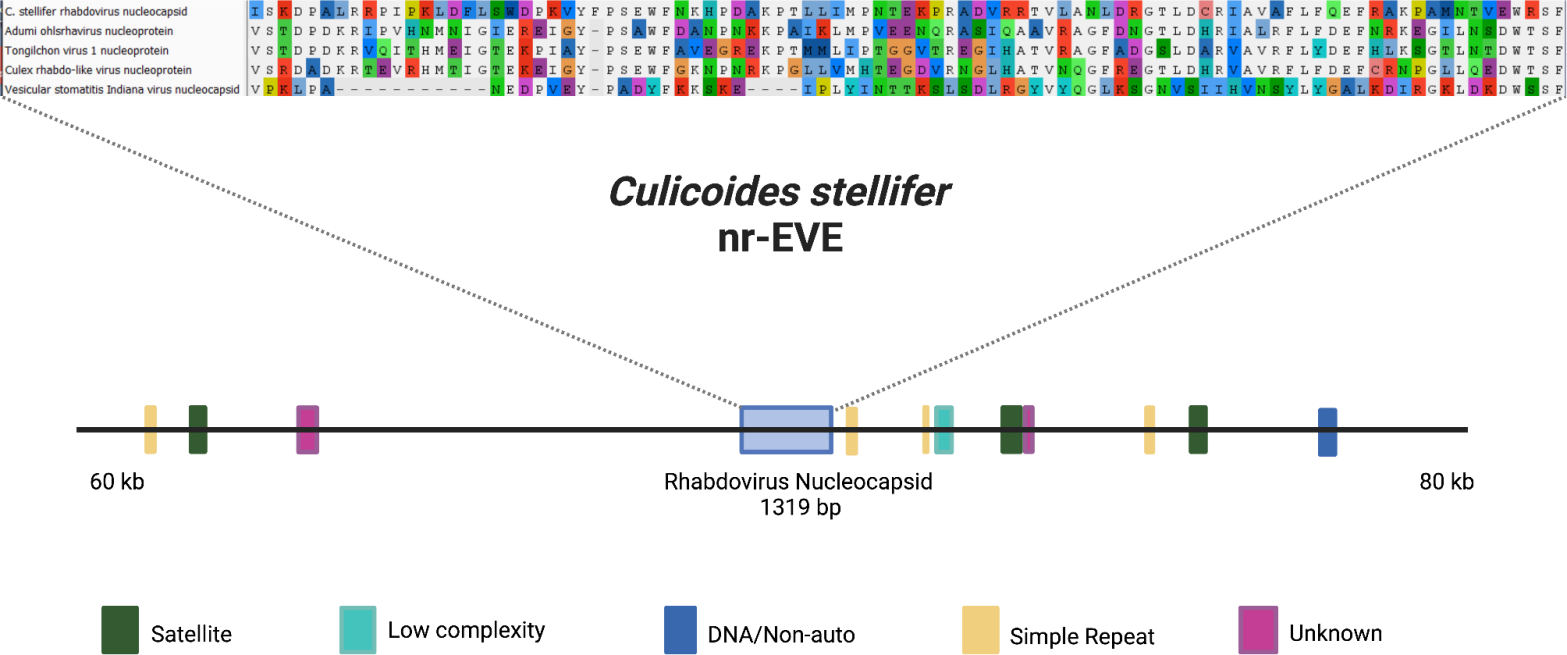
Representation of the non-retroviral endogenous viral element (nr-EVE) sequence found in the *C. stellifer* assembly and the surrounding structural elements in that section of the genome. The sequence is shown aligned to other Rhabdoviruses sequences

The genome query for integrated viral fragments yielded 38 hits, ranging from 44 bp (74.5% identity) to 322 bp (53.2% identity). Fourteen hits greater than 100 bp were queried against the non-redundant protein database in GenBank using blastx. While most of these returned no similar hits or only to RNA-binding domains of genes, a 322 bp fragment in the *C. stellifer* raw reads was found to be similar to VSV. Using blastn we confirmed the presence of this VSV-like fragment in the *C. stellifer* assembly (Fig. 5) and, in conjunction with the gene annotation data, showed that a full 1319 bp coding region for a nucleocapsid was present. A blastx search using this nucleocapsid sequence as a query returned many significant hits (93–98% query coverage, 28.33–38.23% amino acid identity, scores of 161–303, hit length of 1233–1377 bp) to rhabdovirus nucleocapsid proteins in GenBank. To validate the origin of this viral sequence, we mapped the PacBio raw reads to the contig where it is located and found that 17 reads mapped to this contig and that the viral sequence was contained in large high-quality reads.

## Discussion

### Challenges for genomic studies in *Culicoides*

Insect genomics faces challenges in obtaining sufficient high-molecular-weight DNA for high-quality genome assemblies of small-size species. *Culicoides* sizes range from 1 to 3 mm, which makes it very challenging to obtain high-quality genomic DNA. Here, we demonstrated the utility of the ultra-low DNA input PacBio protocol to sequence high-quality reference genomes from a single *Culicoides* individual collected in the field and preserved in ethanol. This opens the door to future biodiversity genomics projects for other small organisms at the millimetre scale. The evidence of some DNA degradation in the sample suggests that fresh frozen insects, or at least fresh-ethanol-preserved specimens kept at -25 °C, will be preferred for future projects. This is essential as the success of the ultra-low DNA input method depends on the quality of the DNA; particularly, the starting amount of biological material correlates with library complexity and is among the factors affecting PCR duplication rate [57].

Despite the limitations associated with PCR amplifications, such as low processivity in high-GC regions, the reduction in overall coverage due to PCR duplicate removal, and PCR-introduced errors, we recovered a genome assembly for *C. stellifer*, with a more complete set of genes identified than in any previous assemblies. This might prove that this workflow can be highly efficient for small and not very complex genomes. The only other genome assembly with higher contiguity was generated using Oxford Nanopore data, which has known problems with base pair accuracy and the potential of sequence errors to confound assembly [58].

Assessing the effect of various levels of duplicate haplotigs purging in combination with two different assembly pipelines was important as insect genomes have high levels of heterozygosity [59]. The tool purge_dups allows the search and removal of false heterotype duplications, which are haplotype sequences that are relatively more divergent than other parts of the genome and are classified as separate genomic regions by the assembly algorithms [60]. The increased contiguity without affecting the overall BUSCO score demonstrates the importance of this step in the data analysis pipeline, as it is highly efficient in purging duplicated regions. Combining long-read sequencing technologies with effective tools to remove duplicates increases the quality of *Culicoides* genome assemblies. In the assembly of *C. sonorensis*, the high level of duplication reported after removing duplicates was likely the result of a misassembly due to heterozygosity in the sample.

### Considerations for genome annotation

The combination of EarlGrey and BRAKER3 for genome annotation resulted in a comprehensive description of the structural elements of the genome. EarlGrey is a pipeline that offers several advantages over other pipelines used for TE annotation. It is specifically designed to enhance TE consensus sequence length and integrity; during curation, almost no elements needed to be substantially adjusted, and RepeatCraft allows it to address issues related to artificial overlapping and fragmented annotations. The landscape of repetitive elements in the genome assembly of *C. stellifer* showed a significant amount of unknown repeats (5,434,496 bp) that are neither satellite DNA nor obvious TEs. A recent study examining 600 insect genomes found that a high percentage of repetitive sequences were not classified in most insect lineages (25-85%). This is mainly associated with reference databases, which have biased representations that impact annotation, particularly affecting insect lineages that have been poorly sampled [61]. As well, for novel genomes it is important to evaluate the taxonomic composition of repeats used in the reference library. The sequencing technology is also an important factor in detecting TE elements. This study reported a 36% increase in the detection of repetitive elements (RE), especially LTRs, when the assembly was generated using long-read sequencing platforms. This highlights the significance of our study in demonstrating the feasibility of the ultra-low input protocol and providing a workflow for genome assembly and annotation of tiny hematophagous flies that serve as vectors of a variety of pathogens. By generating more genomes, we can contribute to insect RE databases and develop the field of RE description as part of biodiversity genomic studies.

The finding of almost no autonomous DNA transposons suggests this genome may be heading to a DNA transposon extinction event in the absence of a horizontal transfer event into the genome, although it is possible that more of the genome remains to be assembled and low copy but autonomous DNA transposons remain in that fraction. Additionally, we may need to apply repeat detection to different assemblies to find lower copy repeats, but this seems challenging given that the few *Culicoides* genomes reported have all been generated with different sequencing technologies and various degrees of completeness and quality. In general, a hierarchical approach of combining repeat libraries from assemblies with different amounts of purged duplicates may be useful if low copy repeats are of interest in any genome project. The most important part of a genome’s structural annotation is the identification of protein-coding genes. We predicted a larger number of proteins in our assembly compared to previously reported genomes [7] (*C. brevitarsis* genome assembly GCF_036172545.1-RS_2024_03), representing about a 20% increase. This can be explained by high accuracy of the genome assembly and the use of software with higher reliability and performance, such as BRAKER3. For *C. sonorensis*, low confidence was reported in 20% of the gene models [62] likely due to problems with the assembly, the gene prediction algorithm or the presence of multicopy gene families.

The lack of transcriptomic data for this species determined that we used clade-specific proteins from OrthoDB as extrinsic evidence to generate hint-guided ab initio gene predictions of protein-coding genes. Identification of the functional role of the proteins found a high percentage of homolog proteins in other organisms (~ 30-55%), with the Swiss Pro database yielding the more comprehensive results.

### Genomic evidence of vector status

The integration of viral genomes (or fragments) into the genomes of their hosts cannot only help us understand evolutionary history and relationships among host species but also offer insights into virus-host interaction [63]. In mosquito genomes, a large number of non-retroviral endogenous viral elements have been detected, and these have been associated with the vector capacity of the species [64]. For example, these can be associated with the production of small RNAs that unfold a response targeting incoming viral transcripts to modulate viral titre, acting as an exogenous antiviral agent that improves the efficiency of the host as an arbovirus vector. In dipterans, the integration of structural viral regions like the nucleoprotein, glycoprotein and matrix regions of the viruses has been more common than non-structural regions integration like the replicase [16].

The virus-midge interaction in *Culicoides* is a complex process that hasn’t been thoroughly studied [65]. Four integrated viral sequences have been reported in *C. sonorensis*, of which three were related to the family *Phasmaviridae* and one to the *Chuviridae*. The hit length ranged from 308 to 998 bp, and the pairwise identity ranged from 25.30 to 35.20% [16]. In dipterans, with the exception of the *Aedes* mosquito genome, in which more than 200 nrEVEs have been identified, a low number of integrated viral sequences have been described (0–1 in *Drosophila melanogaster*, 1 in *Phlebotomus papatasi*, 7 in ​​*Musca domestica*, 5 in tephritid fruit flies, 1–3 in species of *Culicidae* and *Anopheles*) [66]. In tephritid fruit flies, the most abundant nrEVEs reported are *Rhabdoviridae*-derived EVEs, and this was also found for mosquitos [66, 67]. Nevertheless, we consider that an in-depth analysis of nrEVEs in arbovirus vectors is needed and that generating high-quality genome assemblies will be key.

In this study, we identified an nrEVE integrated into the genome of *C. stellifer* that corresponds to the rhabdovirus nucleocapsid proteins, including some matches to VSV. This virus has been previously isolated from single pools of *C. stellifer* during outbreaks in the USA. However, it has never formally been implicated as a vector for VSV [68, 69]. Vesicular stomatitis viruses belong to the family Rhabdoviridae. The genome of VSV has 11,161 nucleotides in length and encodes five major proteins, including the nucleocapsid or ribonucleoprotein. We focused on constructing a library just with the viruses for which *Culicoides* are known vectors with the goal of providing more supporting evidence that *C. stellifer* is a vector of arboviruses. The nrEVE identified is the footprint of a germline viral infection and was then transmitted to the offspring. This finding suggests a close and sustained relationship between rhabdo-like viruses with *C. stellifer* and could indicate that past and present distribution of VSV virus in North America could be linked to this host distribution. Another match corresponds to viruses that have been discovered in culicine mosquitoes (primarily *Ochlerotatus* spp. or *Culex* spp.) from Europe, Asia, Australia, Africa or the Americas. This indicates that we lack enough evidence to fully confirm that this sequence comes from the VSV virus. This can be tackled by sequencing more individuals and improving the completeness of the genome.

The quality of the host genome assembly influences the identification of nrEVEs and was most likely a determinant factor for not finding any arbovirus nrEVE in the genome of *C. sonorensis*. Assemblies based on short-read technology can mask highly repetitive regions where nrEVEs can be found [16]. Additionally, it is important to notice that viruses responsible for an existing nrEVE come from ancient viruses or might have undergone significant mutations over time. In that sense, viral query selection and filtering parameters are important parameters that need to be tuned in for the identification of nrEVEs [66].

## Conclusions

Insects account for the vast majority of eukaryotic biodiversity, and access to genomic resources remains limited for very small metazoans and megadiverse groups. For vector species, like the ones in the genus *Culicoides*, this information is critical for understanding the genetics of virus-host association and the evolution of vector competence in dipterans. Here, we present the first annotated genome of *C. stellifer* from a single specimen using PacBio long-reads. We put forward a workflow to approach data generation and analysis for genome assembly projects focused on tiny insects, paving the way for future improvements that will yield reference genome quality assemblies. This genome has been key in providing further evidence for the vector capacity of *C. stellifer* as we found a nrEVE from the nucleoprotein of a virus from the same family as VSV. The fairly expansive distribution of this species in North America and the potential of a range shift due to climate change requires further investigation as ungulate species in the northern latitudes could be at risk. Increasing the amount of genomic information will play a part in developing a multidisciplinary approach to understanding virus-host interactions and managing viral pathogen transmission to livestock and wildlife.

## Electronic supplementary material

Below is the link to the electronic supplementary material.

Supplementary Material 1

## Data Availability

This genome assembly has been deposited at DDBJ/ENA/GenBank under the accession JBDOCM000000000. The version described in this paper is version JBDOCM010000000. The annotated mitochondrial genome was deposited in GenBank under the accession PP873183.

## Code availability and usage

Data analyses were performed in accordance with the manual and protocols of the bioinformatic tools used. The software version and parameters are outlined in the Methods section. https://github.com/TransposableMan/C-stell-assembly-annotation.git
