# Supplementary material for: Single specimen genome assembly of Culicoides stellifer shows evidence of a non-retroviral endogenous viral element
Source: BMC Genomics. 2025 Mar 14;26:247. doi: 10.1186/s12864-025-11449-5 (PMC11907880; doi:10.1186/s12864-025-11449-5)
Supplement: Supplementary file 1 — Supplementary Material 1 [file 12864_2025_11449_MOESM1_ESM.docx]

| Genome Assembly | *C. stellifer*  HiFiasm  -s 0.35 | *C. stellifer*  HiFiasm  -s 0.35  purge_dups | *C. stellifer*  HiFiasm  -s 0.55  purge_dups | *C. stellifer*  HiFiasm  -s 0.75  purge_dups | *C. stellifer*  IPA | *C. stellifer*  IPA  purge_dups |
| --- | --- | --- | --- | --- | --- | --- |
| Sequencing technology | PacBio Hifi | PacBio Hifi | PacBio Hifi | PacBio Hifi | PacBio Hifi | PacBio Hifi |
| Total length (Mb) | 158 | 119 | 119 | 120 | 146 | 117 |
| Number of contigs | 810 | 450 | 451 | 463 | 730 | 600 |
| Number of scaffolds | 0 | 0 | 0 | 0 | 0 | 0 |
| Longest contig or scaffold (bp) | 1,731,460 | 1,731,461 | 1,731,460 | 1,731,460 | 1,156,022 | 1,156,022 |
| Mean contig or scaffold length (bp) | 194,770 | 265,155 | 264,809 | 259,459 | 199,980 | 195,305 |
| N50 | 356,049 | 479,265 | 459,312 | 458,458 | 289,321 | 306,443 |
| N90 | 84,987 | 132,711 | 132,524 | 113,333 | 104,260 | 98,560 |
| L50 | 126 | 81 | 82 | 83 | 153 | 114 |
| L90 | 476 | 261 | 265 | 269 | 479 | 368 |
| GC content | 30.8% | 30.8% | 30.8% | 30.8% | 30.8% | 30.8% |

Table SI1: Genome assembly statistics of *C. stellifer* using different levels of duplicate removals with HiFiasm.

| BUSCO REPORT | *C. stellifer*  HiFiasm  -s 0.35  Purge_dups | *C. stellifer*  HiFiasm  -s 0.35  Purge_dups |
| --- | --- | --- |
| Datasets | Diptera (diptera_odb10) | Insecta (insecta_odb10) |
| Complete BUSCO | 2953  (89.9 %) | 1332  (97.4 %) |
| Complete single copy | 2882  (87.7 %) | 1291  (3.0 %) |
| Complete duplicated | 71  (2.2%) | 41  (3.0 %) |
| Fragmented | 51  (1.6%) | 7  (0.5 %) |
| Missing | 281  (8.5%) | 28  (2.1 % ) |

Table SI2: BUSCO report for C. stellifer using Diptera and Insecta databases.
